# Methylation of BNIP3 in pancreatic cancer inhibits the induction of mitochondrial-mediated tumor cell apoptosis

**DOI:** 10.18632/oncotarget.18736

**Published:** 2017-06-28

**Authors:** Ye Li, Xu Zhang, Jian Yang, Yi Zhang, Dongming Zhu, Lifeng Zhang, Yanbo Zhu, Dechun Li, Jian Zhou

**Affiliations:** ^1^ Department of General Surgery, The First Affiliated Hospital of Soochow University, Suzhou 215006, China; ^2^ Pancreatic Disease Research Centre, The First Affiliated Hospital of Soochow University, Suzhou 215006, China; ^3^ Department of General Surgery, Suzhou Science & Technology Town Hospital, Suzhou 215006, China; ^4^ Department of General Surgery, Suzhou Hospital Affiliated to Nanjing Medical University, Suzhou 215006, China; ^5^ Department of Oncology, The First Affiliated Hospital of Soochow University, Suzhou 215006, China

**Keywords:** BNIP3, HIF-1α, apoptosis, methylation, pancreatic cancer

## Abstract

Bcl-2 interacting protein 3 (BNIP3) is involved in various cellular processes and is considered a key regulator of hypoxia-induced apoptosis. In the present study, the expression of BNIP3 in pancreatic cancer tissues, the correlation with clinicopathological characteristics and prognosis and the regulation of this protein in pancreatic cancer cell lines with regard to the induction of apoptosis were investigated. BNIP3 expression was significantly lower in pancreatic cancer tissues compared with normal epithelia and was associated with tumor size, clinical stage, and lymph node metastasis. The expression of BNIP3 correlated positively to the proapoptotic protein Bax and negatively to the antiapoptotic protein Bcl-2, whereas the induction of apoptosis by BNIP3 was independent of caspase 3 and 9 activation. The restoration of BNIP3 expression in pancreatic cancer cells *in vitro*, caused loss of ΔΨm, increase in ROS production, and apoptosis induction. The opposite effect was observed in pancreatic cancer cells, following BNIP3 silencing by RNAi. The absence of BNIP3 expression in pancreatic cancer cells was related to gene methylation that suppressed binding of HIF-1α to the BNIP3 promoter, whereas 5-Aza-2′-deoxycytidine (Aza-dC) treatment restored BNIP3 expression and sensitized pancreatic cancer cells to BNIP3-induced apoptosis. The findings indicated that BNIP3 was significantly downregulated in pancreatic cancer resulting in reduced apoptosis induction. Silencing of BNIP3 expression was associated with methylation of the hypoxia-responsive element (HRE) site that in turn inhibited the binding of HIF-1α to the BNIP3 promoter. The data suggest that BNIP3 reactivation is a potential target for therapeutic intervention against pancreatic cancer.

## INTRODUCTION

Human pancreatic cancer exhibits a high malignant potential and poor prognosis and is one of the most lethal malignancies worldwide. It is the fourth cause of carcinoma-associated deaths in western countries, with an overall 5-year survival rate at the range of 2% and 6%. A total of 10% to 20% of the patients present with resectable pancreatic cancer during diagnosis. In addition, recurrence and metastases eventually occur in more than 90% of the patients who undergo surgery [1]. Pancreatic cancer is pathologically characterized by desmoplastic stroma indicating reduced supply of oxygenated blood to the tissues [2]. The ability of the tumor cells to adapt to the hypoxic environment is frequently associated with increased metastatic potential and resistance to chemotherapy. Therefore, the investigation of hypoxia response pathways and the identification of novel targets are of potential therapeutic value.

The incidence of pancreatic ductal carcinoma is associated with deregulation of the proapoptotic and/or antiapoptotic proteins namely, Bcl-2, Bcl-xL, Bax and Bak [3, 4]. Pancreatic cancer cells may acquire tumor resistance to chemotherapeutic agents by downregulation of proapoptotic proteins. Bcl-2 interacting protein 3 (BNIP3) is an atypical Bcl-2 protein family member that contains solely the Bcl-2 homology 3 (BH3) domain. Unlike the other BH3-only members of the Bcl-2 family, BNIP3 interacts with Bcl-2 and Bcl-XL through its transmembrane (TM) domain and N-terminus and not through the BH3 domain [5]. BNIP3 is considered a hypoxia-inducible proapoptotic member of the Bcl-2 family of proteins that has demonstrated differential expression in several types of cancer [6, 7]. Notably in pancreatic cancer, BNIP3 expression is downregulated and this may contribute to the increased resistance of this type of cancer to 5-FU [8].

BNIP3 is involved in various cellular processes and is considered a key regulator of hypoxia-induced apoptosis [9–12]. Induction of BNIP3 overexpression promotes death of a majority of cell types via mitochondrial dysfunction. However, the role of BNIP3 in tumor progression remains controversial. BNIP3 is upregulated in breast cancer [13], lung cancer [14], and follicular lymphomas [15], whereas BNIP3 is not expressed in gastric cancer [16], colorectal cancer [17], and pancreatic cancer [18].

Early studies demonstrated that BNIP3 is regulated by hypoxic conditions. The protein was overexpressed in a variety of cancer cell lines from breast, colorectal, bladder and prostate origin following induction of hypoxia compared with normoxic conditions [7]. Hypoxia plays an important role in tumorigenesis, and tumor cells may increase their survival via the expression of hypoxia-inducible genes. Hypoxia inducible factor 1 (HIF-1), is composed of HIF-1α and HIF-1β and is overexpressed in human malignant tumors [19]. HIF-1α was shown to be involved in the regulation of BNIP3 expression in response to hypoxia [20]. The BNIP3 promoter contains a functional HIF1α responsive element that facilitates binding to the corresponding transcription factor and promotes transcription of the BNIP3 gene under hypoxia [20, 21]. The mechanism of action of BNIP3 involves heterodimerization with the antiapoptotic proteins Bcl-2 and Bcl-xL and subsequent increase of the permeability of the outer mitochondrial membrane pores [5, 20]. This process is independent of cytochrome c release and the activation of caspases [5, 20].

Further studies that investigated the mechanism of BNIP3-mediated apoptosis revealed that this protein activates mitophagy in response to hypoxia [21]. BNIP3 is translocated to the outer mitochondrial membrane and interacts with processed LC3 in order to promote sequestration of mitochondrial degradation via the induction of autophagy [22]. It has been shown that BNIP3-dependent mitophagy restricts the mitochondrial mass and the release of reactive oxygen species (ROS) in growing tumors [22]. Furthermore, glycolysis and angiogenesis are increased in tumors forming in BNIP3 null mice, indicating the negative association of BNIP3 with significant biological processes that are required for tumor cell growth. It has been postulated that HIF1α and BNIP3 exert a novel negative feedback loop and that BNIP3 is inactivated in response to hypoxic conditions [22]. Therefore, mitochondrial dysfunction is promoted in tumors that suppress BNIP3 expression, whereas the activation of mitophagy is impaired by stimuli that would normally induce BNIP3, such as hypoxia and/or nutrient deprivation [22]. The expression of BNIP3 in pancreatic cancer was demonstrated by Okami and colleagues under hypoxic conditions [18]. The expression of the protein was absent in several pancreatic cancer cell lines and it was downregulated in 9 samples of pancreatic adenocarcinoma compared with 8 samples of normal pancreas, due to increased methylation of the BNIP3 promoter [18]. The restoration of BNIP3 expression by treatment of negative BNIP3-pancreatic cancer cell lines with the DNA-methyltransferase inhibitor 5-Aza-2′-deoxycytidine (Aza-dC), rendered the pancreatic cancer cells more sensitive to hypoxia induced cell death [18]. However, the function of BNIP3 and the mechanism of the association of HIF-1α with the BNIP3 promoter in pancreatic cancer cells have not yet been fully elucidated.

In the present study, the expression levels of BNIP3 in human pancreatic cancer tissues and the correlation with clinicopathological characteristics were investigated. In addition, the restoration of BNIP3 expression in the pancreatic cancer BNIP3-negative cell line Capan-1 was examined with regard to the induction of the mitochondrial pathway of apoptosis. The expression of BNIP3 in pancreatic cancer cell lines was further explored in the presence of Aza-dC. Finally, the binding between HIF-1α and the BNIP3 promoter was investigated in association with the methylation status in pancreatic BNIP3-positive cells under hypoxic conditions.

## RESULTS

### BNIP3 expression in pancreatic cancer tissues

**Figure 1:**
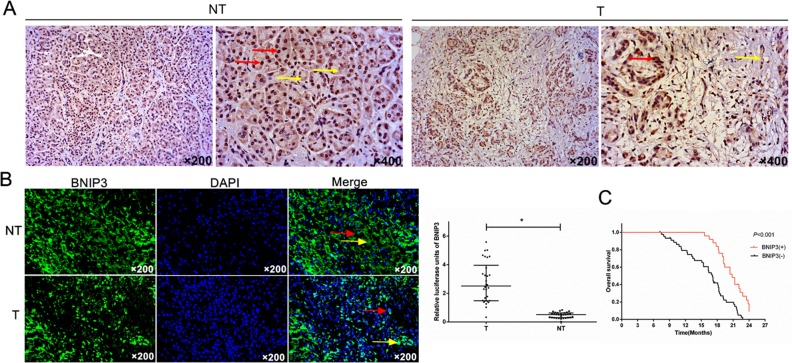
Expression analysis of BNIP3 in pancreatic tumors and prognostic value in pancreatic cancer **(A)** Immunohistochemical staining of BNIP3. IHC indicated that BNIP3 expression was high in adjacent pancreatic non-tumor tissues (NT) and low in pancreatic tumor tissues (T). **(B)** Imunofluorescence staining of BNIP3. IF indicated that BNIP3 protein was predominantly localized to the cytoplasm of cells (yellow arrow), whereas a small proportion of the protein was localized to the nucleus of cells (red arrow). Original magnification: ×200. **(C)** Kaplan–Meier curves of overall survival. BNIP3 negative expression was significantly correlated with an overall shorter survival time period P < 0.001.

The expression of BNIP3 was evaluated in a range of tumor specimens derived from patients with pancreatic cancer by immunohistochemical and immunofluorescence techniques. The clinicopathological characteristics of the tumor samples are summarized in Supplementary Table 1. BNIP3 protein was predominantly localized in the cytoplasm of cells in pancreatic cancer tissues, whereas a lesser amount of the protein was expressed in the nucleus (Figure 1A and 1B). Positive staining was indicated by claybank or brown coloration (Figure 1A). The expression levels of BNIP3 were higher in the non-tumor (NT) tissues compared with the tumor (T) specimens (Figure 1A and 1B). The average fluorescence intensity of BNIP3 for the NT samples was 2.511 (0.326-5.580) compared with 0.510 (0.014-0.837) for the T samples (Figure 1B). The difference in fluorescence intensity was statistically significant (P<0.05).

### Association between BNIP3 expression, clinicopathological parameters and prognosis

A total of 25 out 70 specimens from pancreatic cancer tissues (35.7%) exhibited positive BNIP3 expression, whereas 45 out of 70 non-tumor samples (64.2%) exhibited negative BNIP3 expression (Supplementary Table 1). Significant differences were noted between BNIP3 expression and the parameters tumor stage, N stage and clinical stage (Supplementary Table 1). The remaining parameters namely, age, gender, tumor location and differentiation were not associated with BNIP3 expression, although a tendency towards significance was noted for the parameter age (P=0.064, Supplementary Table 1). The results suggested that low BNIP3 expression correlated with the development of pancreatic cancer.

The survival time in 70 cases of pancreatic cancer following surgery was further compared with regard to BNIP3 expression. The overall survival of the patients with pancreatic cancer and positive BNIP3 expression was higher compared with the patients that were negative for BNIP3 (P<0.001) (Figure 1C). The univariate analysis indicated that patients with lymph node metastasis (P=0.001), clinical stage II (P=0.001) and BNIP3 negative expression (P=0.002) were significantly associated with an increased risk of cancer-related death (Supplementary Table 2). The multivariate analysis revealed that clinical stage (P=0.001) and lymph node metastasis (P=0.002) were key prognostic factors. Furthermore, BNIP3 negative expression (P=0.024) was found to be significantly associated with poor survival in pancreatic cancer patients, irrespective of the clinical stage and lymph node metastasis (Supplementary Table 2). In addition to BNIP3, the expression levels of the antiapoptotic and proapoptotic proteins Bcl-2 and Bax, respectively, were associated with the overall survival of the patients, as demonstrated by univariate and multivariate analyses (P<0.05, Supplementary Table 2). The data indicate that BNIP3 expression is a new prognostic factor in pancreatic cancer.

### Association of BNIP3 expression with proliferation and apoptosis in pancreatic cancer tissue

**Figure 2:**
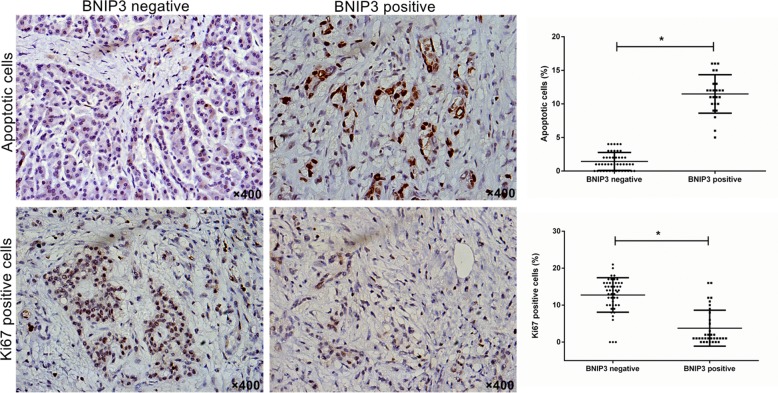
Correlation of BNIP3 expression with proliferation and apoptosis in pancreatic cancer tissues Cell proliferation and apoptosis were examined using Ki67 immunostaining and TUNEL assay, respectively. Original magnification: ×400. *P < 0.05.

The association of the expression of BNIP3 with cellular proliferation and apoptosis was investigated using Ki67 immunostaining and TUNEL assay, respectively. The average number of apoptotic cells was significantly lower in the group that exhibited negative expression of BNIP3 compared with the group that demonstrated positive expression of BNIP3 (P<0.05) (Figure 2). The opposite result was noted for Ki67 (Figure 2). Higher levels of Ki67 expression were noted for the tissues that were stained negative with regard to the expression of BNIP3 compared with those that were stained positive (P<0.05) (Figure 2). The findings suggested that low BNIP3 expression is related to proliferative activity and reduced apoptosis in pancreatic cancer tissue.

**Figure 3:**
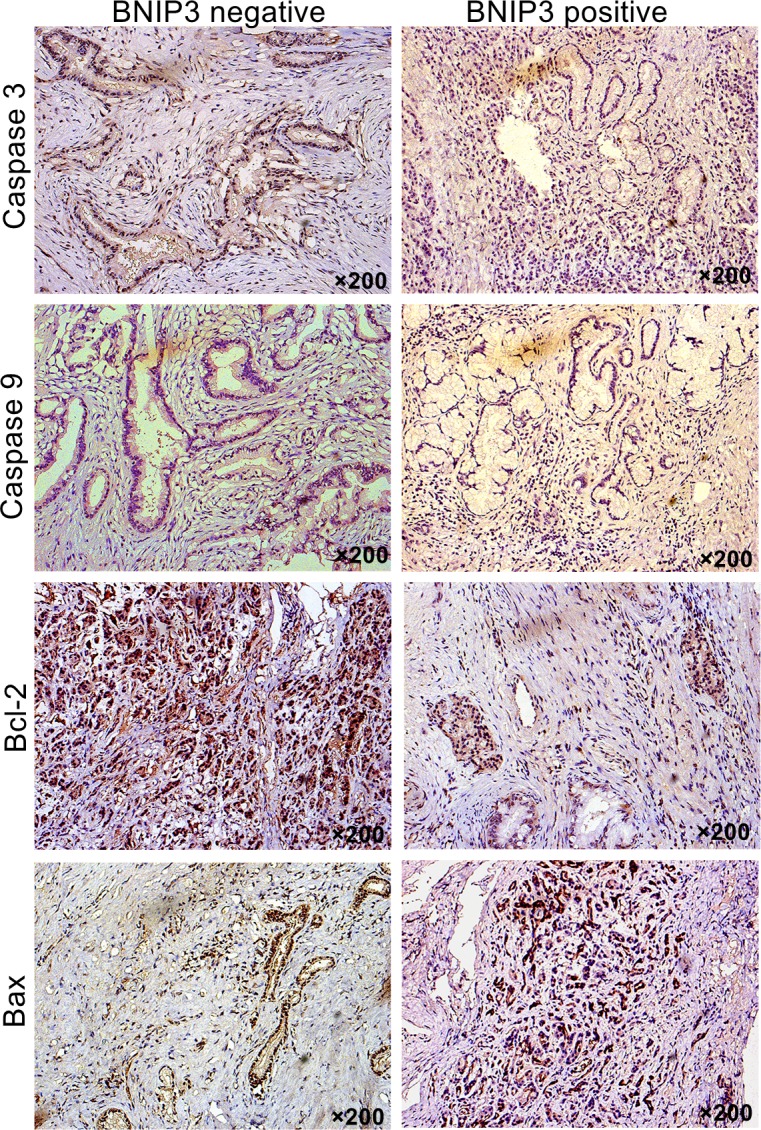
Correlation between BNIP3 expression and apoptosis-associated proteins Expression of Bcl-2, Bax, caspase 3, and caspase 9 were examined via IHC. Original magnification: ×200.

In addition to the aforementioned findings, the positive staining of the apoptosis-associated proteins revealed that their expression was predominantly localized in the cytoplasm of the cells (Figure 3). The positive expression rates of caspase 3, caspase 9, Bcl-2 and Bax proteins were 32.9% (23/70), 27.1% (19/70), 62.9% (44/70), and 42.9% (30/70), respectively. Bcl-2 and Bax expression correlated with OS, as demonstrated by univariate and multivariate analyses (Supplementary Table 2). The proapoptotic marker Bax exhibited higher expression in tissues that were positive for BNIP3 compared with tissues with negative staining (Figure 3). The opposite pattern was noted for Bcl-2 (Figure 3). Bcl-2 expression in the group that revealed negative expression of BNIP3 was statistically different compared with that in the group that demonstrated positive expression of BNIP3 (Supplementary Table 3 P<0.05). The expression of Bax was significantly different between the tissues that were stained positive and/or negative for BNIP3 (Supplementary Table 3, P<0.05). In contrast to Bax, caspase 3 and caspase 9 expression in the two groups exhibited no significant difference (P=0.246 and P=0.324, respectively). Correlation analysis revealed that the expression of BNIP3 was negatively associated with Bcl-2 (r=-0.476, P=0.001) and positively associated with Bax in pancreatic cancer tissues (r=0.333, P=0.005).

### BNIP3 expression in pancreatic cancer cell lines

**Figure 4:**
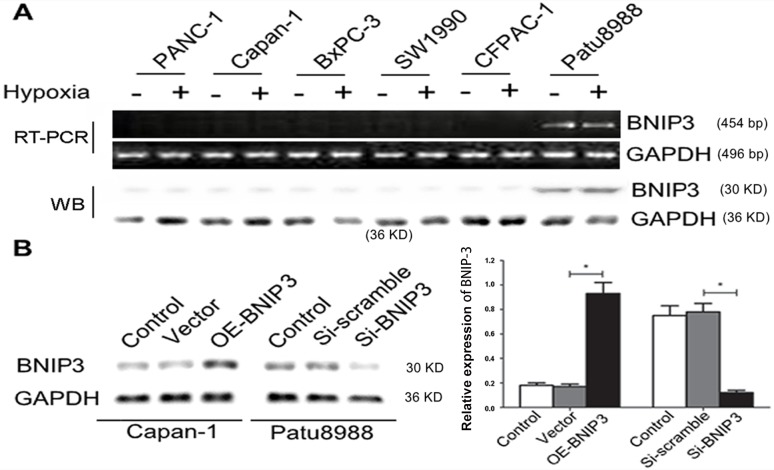
BNIP3 expression in pancreatic cancer cell lines under normoxic and hypoxic conditions **(A)** Relative expression levels of BNIP3 in pancreatic cancer cell lines (PANC-1, Capan-1, BXPC-3, SW1990, CFPAC-1, and Patu8988) under normoxic and hypoxic conditions were measured by RT-PCR and Western blot assays, as demonstrated in materials and methods. **(B)** Ectopic expression and knockdown of BNIP3 in Capan-1 and Patu8988 cells, respectively, as demonstrated by Western blotting. The images are representative of three independent experiments. Data are presented as mean ± SD, *P < 0.05.

The previous studies conducted by our group indicated that BNIP3 expression was significantly lower in pancreatic cancer tissues than in non-tumor tissues. The mRNA and protein expression levels of BNIP3 under normoxic and hypoxic conditions were determined in six pancreatic cell lines namely, PANC-1, BxPC-3, CaPan-1, Patu8988, SW1990, and CFPAC-1. The expression of BNIP3 was undetectable in five of the six pancreatic cancer cells under normoxic conditions, whereas Patu8988 cells revealed low BNIP3 expression (Figure 4A). In addition, hypoxia treatment exerted no effect on BNIP3 expression in the six cell lines (Figure 4A, P>0.05). In order to investigate whether BNIP3 contributes to apoptosis, Patu8988 cells with silenced BNIP3 expression were established by siRNA transfection. Preliminary experiments demonstrated that the BNIP3-si1-homo-587 sequence exhibited the greatest efficiency with regard to the knockdown of BNIP3 expression (Supplementary Figure 1). In contrast to Patu8999 cells, CaPan-1 cells were transfected with a cDNA fragment of BNIP3 in order to overexpress the BNIP3 protein. BNIP3 expression was significantly upregulated in CaPan-1 cells following transfection with OE-BNIP3 (P<0.05), whereas the same protein was significantly downregulated in Patu8988 cells following transfection with si-BNIP3 (P<0.05) (Figure 4B). The relative expression of BNIP3 increased approximately 5-fold following overexpression with the BNIP3 plasmid, whereas it decreased approximately 4-fold following siRNA k/o with si-BNIP3 (Figure 4B).

### BNIP3 induces apoptosis *in vitro* via a mitochondrial pathway

**Figure 5:**
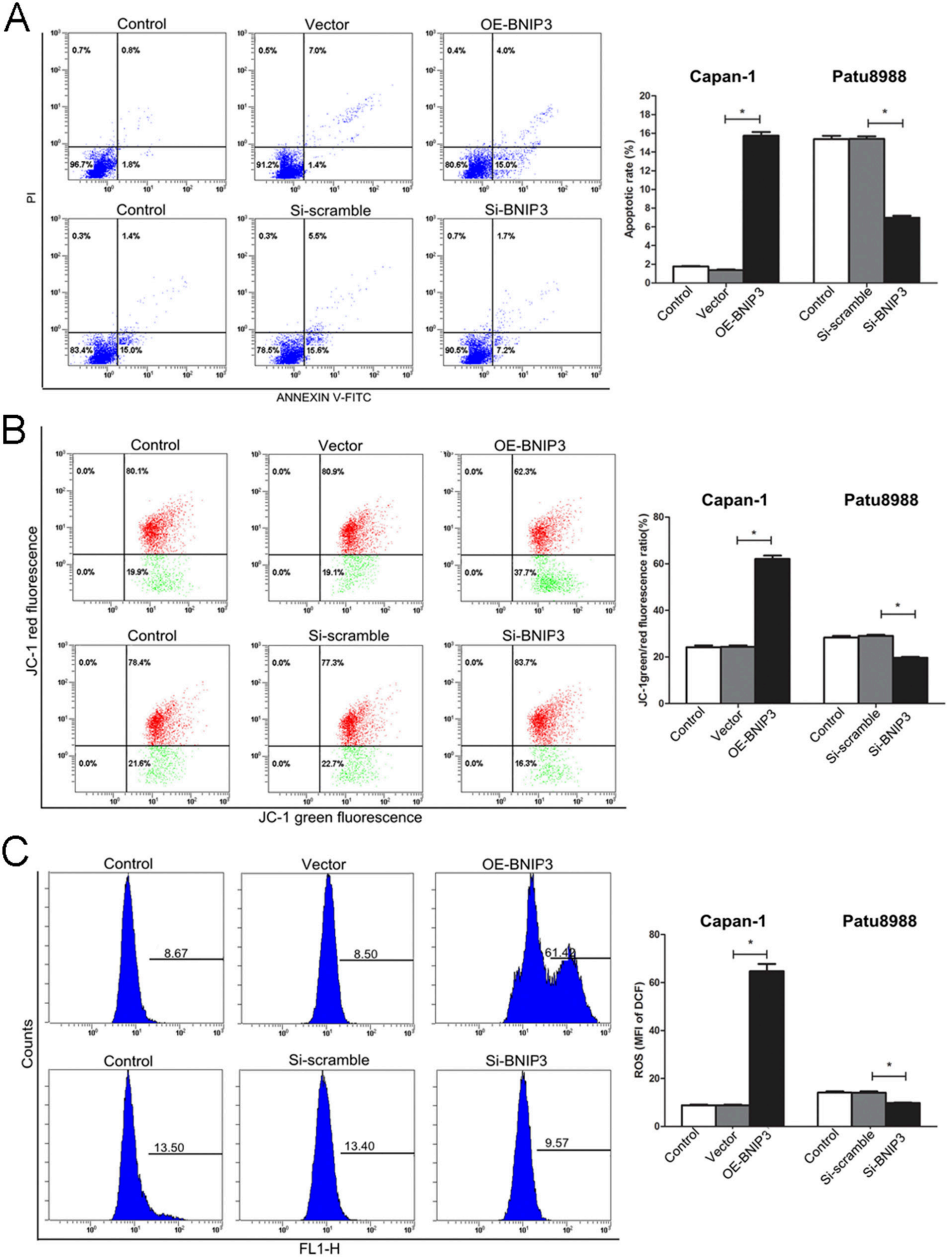
Induction of apoptosis in pancreatic cancer cells by BNIP3 **(A)** Apoptotic cells were labeled with PI/Annexin V, and staining was detected by flow cytometry, as described in materials and methods. **(B)** The changes in the mitochondrial membrane potential were analyzed by green/red fluorescence ratio of JC-1. **(C)** ROS production was monitored in cells incubated with 10 μM of DCFH-DA. Fluorescence intensity was detected by flow cytometry. The images are representative of three independent experiments. Data are presented as mean ± SD, *P < 0.05.

The induction of apoptosis was further investigated in Patu8999 cells that exhibited detectable levels of BNIP3 expression and in the CaPan-1 cell line that was used as a model for overexpression of BNIP3. CaPan-1 OE-BNIP transfected cells enhanced the percentage of apoptotic cells as demonstrated by Annexin V staining, whereas the opposite effect was noted for si-BNIP3 treated cells (Figure 5A). An approximate 8-fold increase in the percentage of apoptosis was noted for the former (15.73%±0.40% vs. 1.37%±0.06%, P<0.05), whereas an approximate 2-fold decrease was noted for the latter (6.97%±0.21% vs. 15.40%±0.26%, P<0.05) (Figure 5A). The function of the BNIP3 protein with regard to the induction of apoptosis was further investigated by measurement of mitochondrial potential (ΔΨm) and ROS. The changes in ΔΨm were analyzed based on the green/red fluorescence ratio of JC-1 (Figure 5B). ΔΨm in the OE-BNIP3 group was markedly higher (approximately 3-fold) compared with that in the vector and control groups (P<0.05), whereas the same parameter was lower (approximately 1.5-fold) following si-BNIP3 treatment compared with the control and siRNA scramble groups (P<0.05) (Figure 5B). The ROS assay indicated similar results, and ROS production was higher in OE-BNIP3 group compared with the vector and control groups (P<0.05), whereas transfection of Patu8988 cells with si-BNIP3 decreased the production of ROS compared to control and siRNA scramble groups (Figure 5C) (P<0.05). The results demonstrated that BNIP3 induces the mitochondrial pathway of apoptosis via the generation of ROS and the loss of ΔΨm.

**Figure 6:**
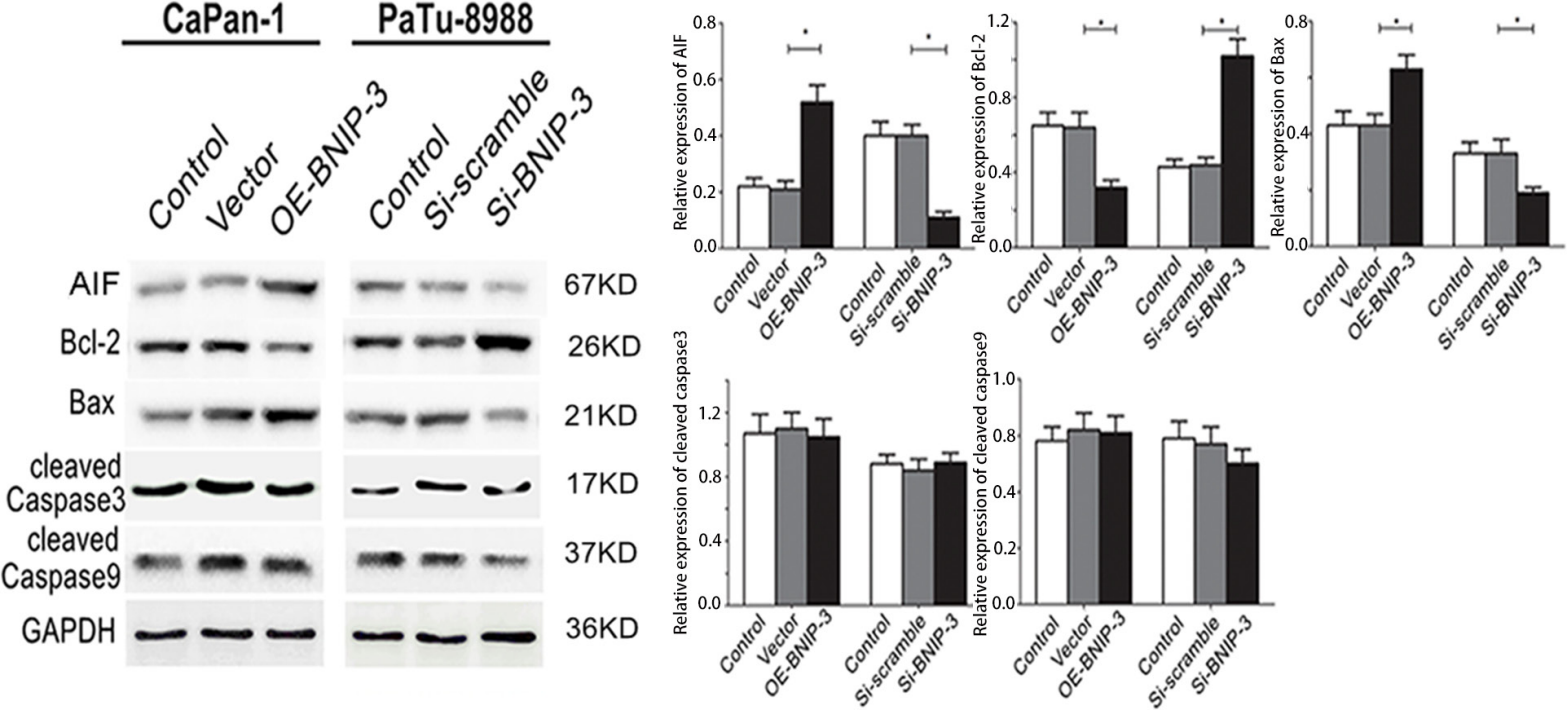
Correlation between BNIP3 and apoptosis-associated proteins in pancreatic cancer cells The expression of apoptosis-associated proteins was examined by Western blot assays following overexpression and/or knockdown of BNIP3. The images are representative of three independent experiments. Data are presented as mean ± SD, *P < 0.05.

The expression of the apoptosis-associated proteins that regulated the expression of BNIP3 was investigated by Western blotting. AIF and Bax were upregulated (P<0.05, respectively), whereas Bcl-2 was downregulated in OE-BNIP3 cells compared with vector and control cells (Figure 6) (P<0.05). Knockdown of BNIP3 protein by si-BNIP3 demonstrated the opposite effect (Figure 6). Therefore AIF and Bax were downregulated, whereas Bcl-2 was upregulated in si-BNIP3 cells compared with control and si-scramble cells (Figure 6) (P<0.05). The regulation of BNIP3 in CaPan-1 and Patu8988 cells exerted minimal effect on the expression of cleaved caspases 3 and 9 (P>0.05 for both). The findings demonstrated that BNIP3 induced apoptosis by the activation of AIF and Bax, and by the suppression of Bcl-2. In addition, BNIP3 induced the mitochondrial apoptotic pathway, which was independent of the expression of caspases 3 and 9 (Figure 6).

### Silencing of BNIP3 gene expression in pancreatic cancer cells by methylation

**Figure 7:**
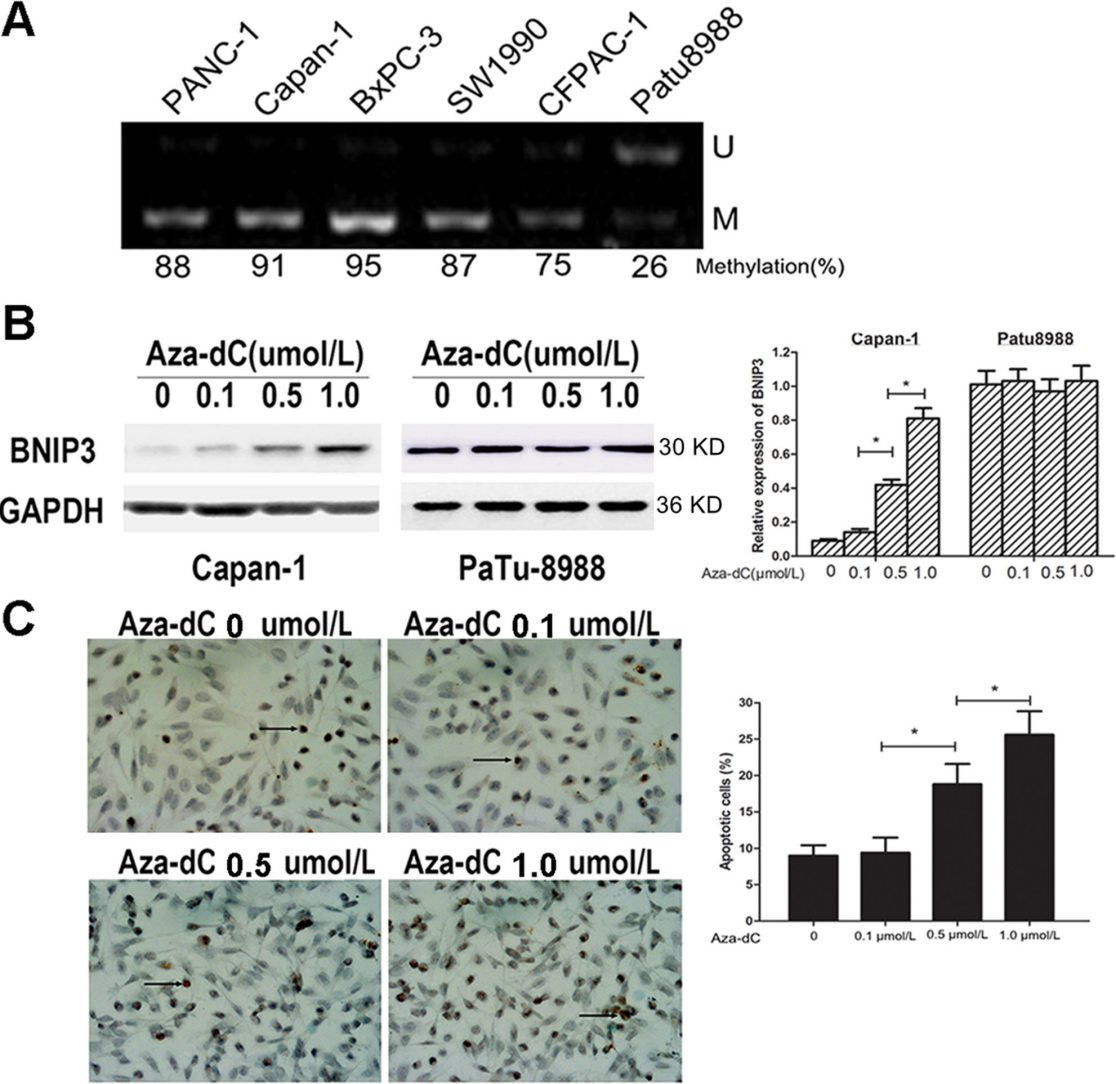
Analysis of BNIP3 methylation and demethylation **(A)** Methylation status of a gene was analyzed by methylation-specific PCR. U: unmethylated gene; M: methylated gene. **(B)** Induction of BNIP3 expression by Aza-dC. The expression levels of BNIP3 in Capan-1 and Patu8988 cells treated with Aza-dC were examined using Western blot assays. Data are presented as mean ± SD of three experiments. *P < 0.05. **(C)** TUNEL assay. The results indicated that the number of apoptotic cells increased which was related to the restored expression of BNIP3 by Aza-dC. Representative images of apoptotic cells captured under an inverted microscope (original magnification: ×400). Data are presented as mean ± SD of five experiments. *P < 0.05.

Previous studies have shown that methylation could silence the BNIP3 gene in tumors via epigenetic mechanisms [18]. In order to confirm the relationship between DNA methylation and BNIP3 silencing, we used MS-PCR to examine the methylation status of the six pancreatic cancer cell lines (Figure 7A). A total of five out of the six pancreatic cancer cell lines indicated increased methylation levels in the promoter region of the BNIP3 gene that were associated with silencing of BNIP3 expression, whereas Patu8988 cells that exhibited low BNIP3 expression displayed partial methylation (26%) (Figure 7A). The expression of BNIP3 following treatment with the methyltransferase inhibitor Aza-dC (Figure 7B) was further examined. Treatment of Capan-1 cells with 0.1, 0.5 and 1 μM of Aza-dC resulted in a dose dependent increase (P<0.05 for 0.5 and 1 μM) in the expression of BNIP3 compared to solvent control (0 μM), whereas the same treatment in Patu8988 cells did not exhibit a significant change in BNIP3 expression levels (Figure 7B). TUNEL assay further demonstrated that the increase in BNIP3 expression following 0.5 and 1 μM of Aza-dC treatment was associated with the induction of apoptosis (Figure 7C). A an approximate 2.5-fold increase in the apoptotic population of the cells was demonstrated at 1 μM of Aza-dC treatment that in turn caused a 6-fold increase in the protein levels of BNIP3 compared with solvent control cells (Figure 7B and 7C). The findings indicated that BNIP3 silencing in pancreatic cells was related, at least in part, to gene methylation. Demethylation by Ada-dC could restore BNIP3 expression and sensitize pancreatic cancer cells to BNIP3-induced apoptosis.

### Suppression of binding between HIF-1α and the BNIP3 promoter by methylation

Based on the previous findings regarding the methylation pattern and the expression of BNIP3 under hypoxic conditions in Patu8988 cells, the association between the BNIP3 promoter and the transcription factor HIF-1α was investigated. The expression of BNIP3 and HIF-1α proteins was investigated under normoxic and hypoxic conditions in the six pancreatic cancer cell lines (Supplementary Figure 2A). HIF-1α expression levels were markedly increased in all six pancreatic cancer cell lines following induction of hypoxia (Supplementary Figure 2A). In contrast to HIF-1α, BNIP3 protein expression levels were undetectable in five out of six cell lines, and only Patu8988 cells exhibited low BNIP3 expression that was not affected by the normoxic and/or hypoxic conditions (Supplementary Figure 2A). A previous study demonstrated that HIF-1α could activate BNIP3 by binding to the BNIP3 promoter [20]. However, this phenomenon was not observed in the present study. Sequence analysis of the BNIP3 gene promoter, indicated that a CpG island in the promoter spans from −660 bp to +1 bp of the transcription start site, where abnormal methylation frequently occurs (Supplementary Figure 2B). In addition, the BNIP3 gene promoter contains a hypoxia-responsive element (HRE) located at −206 bp, which is required for binding to the transcription factor HIF-1α as previously reported [5]. In order to determine whether HIF-1α is associated with BNIP3 expression due to the methylation of the HRE region, ChIP assays on Aza-dC-treated Capan-1 cells were conducted. Following treatment with Aza-dC at 1 μM, the binding of HIF-1α to the BNIP3 promoter significantly increased, whereas no binding was observed in untreated cells (Supplementary Figure 2C). This finding suggested that methylation suppressed the binding between HIF-1α and the HRE site at the BNIP3 promoter, inducing silencing of BNIP3 expression in pancreatic cancer cells despite the presence of HIF-1α.

## DISCUSSION

In the present study, the contribution of the proapoptotic protein BNIP3 in the induction of apoptosis in pancreatic cancer was examined. BNIP3 was downregulated in pancreatic tumor tissues compared with the corresponding normal specimens, whereas the expression of this protein was absent in the pancreatic cancer cell lines investigated, with the exception of Patu8988 cells. The expression of BNIP3 was associated with increased apoptosis, decreased cellular proliferation, increased Bax and decreased Bcl-2 levels in the tumor tissues. It is important to note that the induction of apoptosis in BNIP3-positive cells and/or tissues was mediated via the mitochondrial pathway in the absence of the activation of caspases 3 and 9. Furthermore, the expression of BNIP3 in the cancer cell lines appeared to be associated with the methylation status of the cells, while in the presence of the DNA-methyltransferase inhibitor Aza-dC the restoration of BNIP3 expression was achieved in Capan-1-BNIP3-negative cells and this process was accompanied by increased induction of apoptosis. Finally, the transcriptional activation of BNIP3 was proven to be mediated by direct binding of the BNIP3 promoter region to HIF1-α in the presence of Aza-dC. Taken collectively, the data suggest that HIF1-α mediates activation of BNIP3 under hypoxic conditions that results in the induction of the mitochondrial pathway of apoptosis in pancreatic cancer cells, via the modulation of the expression of the proteins Bax, AIF and Bcl-2. It is important to note that the binding of HIF1-α to the HRE BNIP3 promoter region is inhibited by methylation, thus affecting the expression of BNIP3 in pancreatic cancer cells.

The expression of BNIP3 has been previously investigated in pancreatic tissues and it was found that this protein is differentially expressed in pancreatic ductal adenocarcinoma, as determined by a combination of microarray and RT-PCR methodologies [23]. BNIP3 was documented as a clinically important diagnostic and prognostic biomarker [23]. Although BNIP3 expression is elevated in certain tumors namely, breast, lung and cervix in colorectal and pancreatic cancers BNIP3 is frequently epigenetically silenced, possibly reflecting the different functions of BNIP3 according to the different tissues [24]. The findings of the present study are in concordance with the study by Okami and colleagues, where it was found that BNIP3 was downregulated in pancreatic tumors (n=9) compared with normal tissues (n=8) by RT-PCR analysis [18]. Mahon et al. reported similar findings as the expression of BNIP3 was shown to be reduced in 20 samples of pancreatic ductal adenocarcinoma compared with normal epithelia by immunohistochemichal techniques [25]. The results reported in the current study advance further to include a larger sample size of tumors compared with the studies conducted by Mahon et al. and Okami et al. In addition, the expression of BNIP3 exhibited a positive correlation with the induction of apoptosis and the induction of the apoptotic protein Bax, whereas a negative correlation was noted for the anitapoptotic protein Bcl-2 in the tumor tissues. The expression of BNIP3 indicated no correlation with the expression of caspases 3 and 9. To the best of our knowledge, this is the first study that has demonstrated a correlation between the expression of BNIP3 and the apoptotic protein markers in pancreatic cancer.

In addition to the expression analysis of BNIP3 in pancreatic tumors, the results revealed that BNIP3 expression was associated with clinicopathological characteristics, including tumor size, clinical stage, and lymph node metastasis. BNIP3 negative expression was significantly associated with an increased risk of cancer-related death. Univariate and multivariate analysis of survival indicated that the level of BNIP3 expression is an independent prognostic indicator in pancreatic cancer. These findings are in agreement with the study conducted by Erkan et al. where the expression of BNIP3 was investigated in a panel of 26 pancreatic ductal adenocarcinoma tissues and compared with the overall survival of pancreatic cancer patients [26]. BNIP3 positive patients exhibited higher survival compared with BNIP3 negative patients that is in concordance with the present study [26]. This is probably due to the reduced sensitivity of the tumor cells that were BNIP3 negative to the induction of apoptosis. Consequently, patients with pancreatic cancer that exhibit higher expression of BNIP3 possess a higher overall survival rate.

The mechanism of BNIP3-mediated apoptosis in pancreatic cancer was further investigated in *in vitro* cell lines under hypoxic and normoxic conditions. Previous studies have reported that BNIP3 expression can be induced under hypoxia in certain pancreatic cancer cell lines namely, PSN-1, T3M4, PANC-1, HPDE, CFPacl and SU86.86 [18, 25, 26]. In the present study, detectable BNIP3 expression was noted solely in Patu8988 cells, whereas in the cell lines PANC-1, Capan-1, BxPC-3, SW1990 and CFPAC-1 the expression of the BNIP3 mRNA and/or protein was absent. The expression of BNIP3 in the PANC-1 cell line as demonstrated in the present study is contradictory to the studies by Erkan et al and Mahon et al., whereas it is in agreement with the study conducted by Okami et al. [18, 25, 26]. The possible explanations for these discrepancies are the differences in the sensitivity of the detection techniques used in each study, the quantification assays of the expression levels of BNIP3 and the culture conditions used. The contribution of hypoxia in the induction of BNIP3 has been previously documented [27]. It was proposed that the hypoxic environment promoted an aggressive phenotype of pancreatic cancer cells that were BNIP3 negative and remained resistant to future BNIP3 induction [26]. The downregulation of BNIP3 under hypoxic conditions may provide an advantage towards the increased survival of the tumor cells, whereas patients with low BNIP3 levels are expected to exhibit lower survival rate [26]. The early hypoxic induction of BNIP3 possibly aids the selection of apoptosis resistant clones within the hypoxic region leading to the more aggressive malignant phenotype in the later stages [28].

The initial findings derived from of the clinical tissues with regard to BNIP3 expression and induction of apoptosis were confirmed by *in vitro* cell line models, since knockdown of BNIP3 resulted in reduced apoptosis, whereas overexpression of BNIP3 enhanced the population of apoptotic cells. The induction of apoptosis was mediated via a mitochondrial Bax/Bcl-2-pathway, yet in the absence of the activation of caspases 3 and 9. BNIP3 downregulation has been shown to enhance the resistance to 5-FU in pancreatic cancer cell lines suggesting that the expression of this protein is essential for the induction of tumor cell death [26]. Furthermore, knockdown of the transcription factor S100A4 enhances apoptosis of pancreatic cancer cells in response to gemcitabine via the induction of BNIP3 expression [25]. Previous investigations revealed that BNIP3 promotes mitochondrial membrane permeabilization through activation of the Bax/Bak pathway [29]. The activation of BNIP3 that initiates opening of the mitochondrial permeability transition pore and cell death in the absence of caspase activation has been previously reported in neonatal cardiac myocytes but not in pancreatic cancer cells [30]. The mechanism of action was notably attributed to the suppression of caspase 3 and 9 expression by the calcium-dependent proteins calpains [30]. During hypoxia-acidosis the calpain-dependent cleavage of procaspase 3 prevents the activation of caspase 3 in cardiac myocytes, suggesting that cardiac myocyte loss caused by hypoxia-acidosis is caspase-independent but BNIP3-, and mitochondrial transition pore-dependent [30]. It was suggested that the low pH stabilized BNIP3 and promoted the integration of the protein into the mitochondrial membranes where it caused the opening of the mitochondrial transition pores [30]. The aforementioned findings may explain the involvement of the caspase 3 and 9-independent induction of apoptosis by BNIP3 in pancreatic cancer cells. In addition to Bax, the protein AIF, a proapoptotic protein present in the intermembrane of mitochondria, was induced following overexpression of BNIP3 and downregulated following BNIP3 knockdown. Increased AIF expression induces sensitivity to cell death, whereas AIF silencing provides protection against apoptosis [31].

The methylation analysis conducted in the present study demonstrated that the cell lines that did not express BNIP3 were highly methylated, whereas inhibition of methylation in the Capan-1-BNIP3-negative cell line restored BNIP3 expression that was parallel with the induction of apoptosis. Multiple factors namely, DNA methylation, histone deacetylation, and transcription factor recruitment have been shown to repress BNIP3 expression [32–35]. Hypermethylation in the BNIP3 promoter occurs in a majority of tumors, including pancreatic cancer [18], hepatocellular carcinoma [36], colon cancer [35], and gastric cancer [37]. The results reported in the present study are in concordance with the study by Abe and coworkers that demonstrated no BNIP3 expression in 6 out of 12 pancreatic cancer cell lines under normoxic conditions, due to DNA methylation of the CpG island of BNIP3 gene in the region around the transcription start site [38]. The expression of BNIP3 was restored by the methyltransferase inhibitor 5-aza-deoxycytidine (5-aza-dC), as was the hypoxia-mediated pancreatic cancer cell death [38]. Taken together the findings indicate that the reduced BNIP3 expression in pancreatic cancer results from epigenetic silencing at the BNIP3 promoter region due to methylation. This conclusion is supported by previous studies [32, 39].

The present study further demonstrated that methylation occurred at a CpG island including the HRE region of the BNIP3 promoter and inhibited the binding of HIF-1α to the HRE region. HIF-1α is one of the most important transcription factors that are activated under the hypoxic microenvironment. BNIP3 is a HIF1α target that is induced by hypoxia but is also regulated by a variety of additional transcription factors namely, RB-E2F1, TP53, FOXO3 and NF-κB [22]. HIF-1α and BNIP3 are reported to be overexpressed in hypoxic regions [15]. The present study demonstrated that HIF-1α expression was significantly induced in pancreatic cancer cells cultured under hypoxic condition, whereas BNIP3 expression was undetectable. This result suggested that BNIP3 activation was disrupted following HIF-1α activation in pancreatic cancer cells. However, to the best of our knowledge, the mechanism of HIF-1α-mediated BNIP3 induction in pancreatic cancer cells has not yet been fully elucidated. It is important to note that the contribution of additional transcription factors may be involved in the transcriptional activation of BNIP3 by HIF-1α in pancreatic cancer cells, as earlier noted for other types of cancers [40, 41]. For example, the member of the basic helix-loop-helix/PAS (bHLH/PAS) transcription factors SIM2s, has been shown to attenuate BNIP3 hypoxic induction via cross-talk with HIF-1α on the HRE in prostate cancer cells [41]. The functional interference of SIM2s with the activity of HIF1α on BNIP3 may be involved in the hypoxic regulation of BNIP3 in pancreatic cancer cells, although further studies are required to confirm this hypothesis.

The present study demonstrated that BNIP3 was significantly downregulated in pancreatic cancer and this correlated with a poor prognosis. BNIP3 acted as a proapoptotic protein in pancreatic cancer cells and induced apoptosis via the mitochondrial pathway. Silencing of BNIP3 expression was associated with methylation of the hypoxia-responsive element site that inhibited binding of HIF-1α to the BNIP3 promoter. The data collectively suggested that BNIP3 reactivation is a novel therapeutic target for pancreatic cancer.

## MATERIALS AND METHODS

### Clinical data

Tissue samples, including tumor tissues and adjacent non-tumor tissues, were obtained from 70 patients with pancreatic cancer during surgical resections conducted at the First Affiliated Hospital of Soochow University between January 2010 and December 2014. The tissue samples were immediately frozen in liquid nitrogen following surgical removal and further stored at −80°C.

The inclusion criteria were as follows: All enrolled pancreatic cancer patients were confirmed by pathological examination; patients were eligible for radical surgery, due to the incidence of pancreatic cancer. T1 and T2 stage patients with radical resection were enrolled.

The exclusion criteria were as follows: Patients who received preoperative chemo-, radio-, or immunotherapy were excluded.

Patients with pancreatic cancer were followed up once every 3 months in out-patient department for a total period of 2 years. A total of 4 cases were lost during the follow-up period.

All samples were classified in accordance with the tumour node metastasis (TNM) classification and criteria of the World Health Organisation (WHO), whereas tumour grade was assessed according to the WHO criteria. The present study was approved by the ethics committee of the First Affiliated Hospital of Soochow University.

### Cell culture

A total of six pancreatic cancer cell lines (Panc-1, Capan-1, BxPC-3, SW1990, CFPANC-1, and Patu8988) were obtained from the Chinese Academy of Sciences (Shanghai, China). The cells were cultured in high-glucose DMEM (HyClone, China) containing 10% fetal bovine serum (FBS), 100 units/ml penicillin, and 100 μg/ml streptomycin. The cells were subjected to hypoxic conditions that comprised 1% O_2_, 5% CO_2_, and 94% N_2_ at 37°C, as described in previous studies [10, 38].

### Expression plasmids and BNIP3 gene silencing

A 660-bp cDNA fragment of BNIP3 was synthesized and cloned into a pEX-2 plasmid (OE-BNIP3) by Gene Pharma Co., Ltd. (Shanghai, China) for the construction of the eukaryotic expression vectors. Empty pEX-2 vectors served as negative control (vector). Small interfering RNA (siRNA) was constructed by Gene Pharma Co., Ltd. (Shanghai, China). A total of three different siRNA sequences were designed for BNIP3 targeting. The sequences were the following:

BNIP3-si1-homo-222 sense (5‘–3‘) CAGCCUCG GUUUCUAUUUATT, antisense (3‘–5‘) UAAAUAGA AACCGAGGCUGTT, BNIP3-si1-homo-587 sense (5‘–3‘) GGCAUAUUCUCUGCAGAAUTT, antisense (3‘–5‘) AUUCUGCAGAGAAUAUGCCTT, BNIP3-si1-homo- 511 sense (5‘–3‘) CCCAAGGAGUUCCUCUUUATT, antisense (3‘–5‘) UAAAGAGGAACUCCUUGGGTT.

A nonspecific scrambled siRNA sequence (si-Scramble) was used as negative control (target sequence: 5′-UUCUUCGAACGUGUCACGUTT-3′). Capan-1 and Patu8988 cells that expressed OE-BNIP3 or si-BNIP3 were established using Lipofectamine 2000 (Invitrogen, USA).

### Quantitative real-time polymerase chain reaction (qRT-PCR)

Total RNA was extracted from tissues and cells using Trizol reagent (Invitrogen, USA). Single-strand cDNA was synthesized from 10 μg of total RNA using random primers and M-MLV reverse transcriptase (Gibco-BRL, USA). BNIP3 and GAPDH mRNA transcript levels were assessed by qRT-PCR using the following primers: BNIP3, F: 5′-GCCCACCTCGCTCGCAGACAC-3′ and R: 5′-CAATCCGATGGCCAGCAAATGAGA-3′; GAPDH, F: 5′-AGCGAGCATCCCCCAAAGTT-3′ and R: 5′-GGGCACGAAGGCTCATCATT-3′. PCR reactions were carried out for 40 cycles that comprised a denaturation step at 95°C for 15 s, an annealing step at 62°C for 45 s and an extension step at 72°C for 30 s. The amplified segments were analyzed using gel electrophoresis.

### Drug experiment

The methyltransferase inhibitor Aza-dC (Sigma, USA) was used to reverse methylation. The cells were treated with Aza-dC at final concentrations of 0.1, 0.5, and/or 1.0 μM and co-cultured for 3 days prior to the analysis. Untreated controls were prepared in parallel by addition of an equal volume of DMSO.

### Apoptosis assay

A total of 100 μl of cell suspension (1×10^6^ cells/ml) was labeled with 10 μl of PI and 5 μl of Annexin V/FITC (BD Biosciences, USA) in order to detect the percentage of the apoptotic cells. The cells were incubated in the dark for 15 min at room temperature, and apoptosis was assessed by a FACS Calibur flow cytometer.

### Reactive oxygen species (ROS) detection and mitochondrial membrane potential (ΔΨm) assay

The production of ROS was monitored by the compound 2′, 7′-dichlorofluorescin diacetate (DCFH-DA). A total of 1×10^6^ cells/ml were harvested, washed three times with ice-cold PBS, and stained with 10 μmol/l DCFH-DA (Sigma, USA) at 37°C for 20 min. Fluorescence intensity was detected by flow cytometry.

The ΔΨm assay was conducted using a mitochondria-specific cationic dye (JC-1, Sigma, USA). Cells were incubated in fresh culture media containing 2.5 μg/ml JC-1 for 30 min at 37°C in the dark. The analysis was conducted using flow cytometry.

### Methylation-specific PCR (MS-PCR)

The methylation status of the gene promoters was analyzed by MS-PCR. The primers were designed to contain four or five CpG dinucleotides in order to detect differences in the sequences of methylated and unmethylated DNA, following bisulfite treatment. The primers for the unmethylated reaction were as follows: FP 5′-TAGGATTTGTTTTGTGTATG-3′ and RP 5′-ACCACATCACCCATTAACCAC

A- whereas for the methylated reaction, the primers were the following: FP 5′-TAGGATTCGTTTCGCG TACG-3′ and RP 5′-ACCGCGTCGCCCATTAAC CGCG-3′. The PCR conditions described previously were used in the present study [18].

### Western blot assay

Total proteins were separated by 10% SDS-PAGE and blotted onto PVDF membranes. The membranes were blocked with 10% non-fat milk powder at room temperature for 2 h and incubated with the following primary antibodies: anti-BNIP3 (1:500), anti-HIF-1α (1:1000), anti-AIF (1:1000), anti-Bcl-2 (1:1000), anti-Bax (1:1000), anti-caspase 3 (1:1000), anti-caspase 9 (1:1000) (all obtained from Abcam, UK) and anti-GAPDH (1:1000, Santa Cruz Biotechnology, USA) at 4°C overnight. Following three washes with PBS-T (PBS with 0.05% Tween-20), the membranes were incubated with a horseradish peroxidase-conjugated goat anti-mouse immunoglobulin G (IgG; 1:2,000; Santa Cruz Biotechnology, USA). Reactive bands were detected using ECL Western Blotting Detection Reagent (GE Healthcare, USA).

### Chromatin immunoprecipitation (ChIP) assay

ChIP assay was carried out using a ChIP assay kit (Upstate Biotechnology, USA) according to the instructions provided by the manufacturer. The immunoprecipitation was conducted with an anti-HIF-1α monoclonal antibody (Abcam, UK) and/or mouse IgG (negative control). The PCR primers for the binding of HIF-1α to the BNIP3 promoter were as follows: FP 5′-CGCGCCGCACGTGCCACA-3′ and RP 5′-TGTGGGCACGTGCGGCGCG-3′ (196 bp).

### TUNEL assay

The cells were seeded in cell culture slides (Becton Dickinson, USA), treated with Aza-dC at final concentrations of 0.1, 0.5, and/or 1.0 μM and subsequently allowed to grow for three days. The slides were washed with PBS, fixed for 15 min with 10% neutral buffered formalin and evaluated using the TUNEL System Kit (Roche, Swiss) according to the instructions provided by the manufacturer. The apoptotic cells were calculated by counting the positive cells in five visual fields under an optical microscope at × 200 magnification.

### Immunohistochemistry (IHC) and immunofluorescence (IF)

Serial sections (4 μm) that were subjected to immunohistological staining were fixed with fresh solution of 3% H_2_O_2_ that contained 0.1% sodium azide in order to block endogenous peroxidase activity. Following incubation with antigen retrieval solution and blocking reagent for 15 and 20 min respectively, the sections were incubated overnight at 4°C with primary anti-BNIP3 (1:500), anti-Bcl-2 (1:1000), anti-Bax (1:1000), anti-caspase 3 (1:1000) and/or anti-caspase 9 (1:1000) antibodies (all from Abcam, UK). The immunostaining intensity and the staining percentage of BNIP3 expression was evaluated by light microscopy, following 2 h incubation with a HRP IgG secondary antibody (1:2000; Santa Cruz Biotechnology, USA). A total of five fields of view were examined at ×200 magnification. The staining intensity was scored as follows: 0, no staining; 1, weak staining; and 2, moderate to strong staining. The percentage of positively stained cells was scored as follows: 0, <10%; 1, 10%–50%; and 2, >50%. The final score was the sum of the intensity score and quantity score. A score of >2 indicated positive expression. In order to determine the expression of Ki-67, positively stained cells were defined as those that exhibited clear nuclear staining. The tissues were considered Ki67 positive, when >15% of the ≥1,000 tumor cells were stained.

The immunofluorescent staining of BNIP3 expression was observed under a fluorescence microscope following incubation of the samples with 4′, 6-diamidino-2-phenylindole (DAPI) for 20 min at room temperature in the dark.

### Statistical analysis

All data were evaluated using the SPSS software version 19.0. The data are presented as mean ± SD. Continuous variables were compared using one-way analysis of variance (ANOVA) followed by SNK post hoc and Mann-U-Whitney tests. Categorical variables were compared by the Chi-square (χ^2^) test. Correlation analysis was carried out using Spearman analysis. The survival curves were calculated using the Kaplan–Meier method and the differences were assessed by a log-rank test. Cox regression analysis was used to determine the independent factors, which were based on the variables selected by a univariate analysis. A P value of less than 0.05 (P<0.05) was considered statistically significant.

## SUPPLEMENTARY MATERIALS FIGURES AND TABLES



## Abbreviations

BNIP3: Bcl-2 interacting protein 3
HRE: hypoxia-responsive element
BH3: Bcl-2 homology 3
TM: transmembrane
ROS: reactive oxygen species
NT: non-tumor
TNM: tumour node metastasis
WHO: World Health Organisation
FBS: fetal bovine serum
siRNA: Small interfering RNA
qRT-PCR: Quantitative real-time polymerase chain reaction
MS-PCR: Methylation-specific PCR
ChIP: Chromatin immunoprecipitation
IHC: Immunohistochemistry
IF: immunofluorescence
