# Supplementary material for: Methylation of BNIP3 in pancreatic cancer inhibits the induction of mitochondrial-mediated tumor cell apoptosis
Source: Oncotarget. 2017 Jun 28;8(38):63208–22. doi: 10.18632/oncotarget.18736 (PMC5609914; doi:10.18632/oncotarget.18736)
Supplement: Supplementary file 1 [file oncotarget-08-63208-s001.pdf]

Methylation of BNIP3 in pancreatic cancer inhibits the induction of mitochondrial-mediated tumor cell apoptosis

SUPPLEMENTARY FIGURES AND TABLES

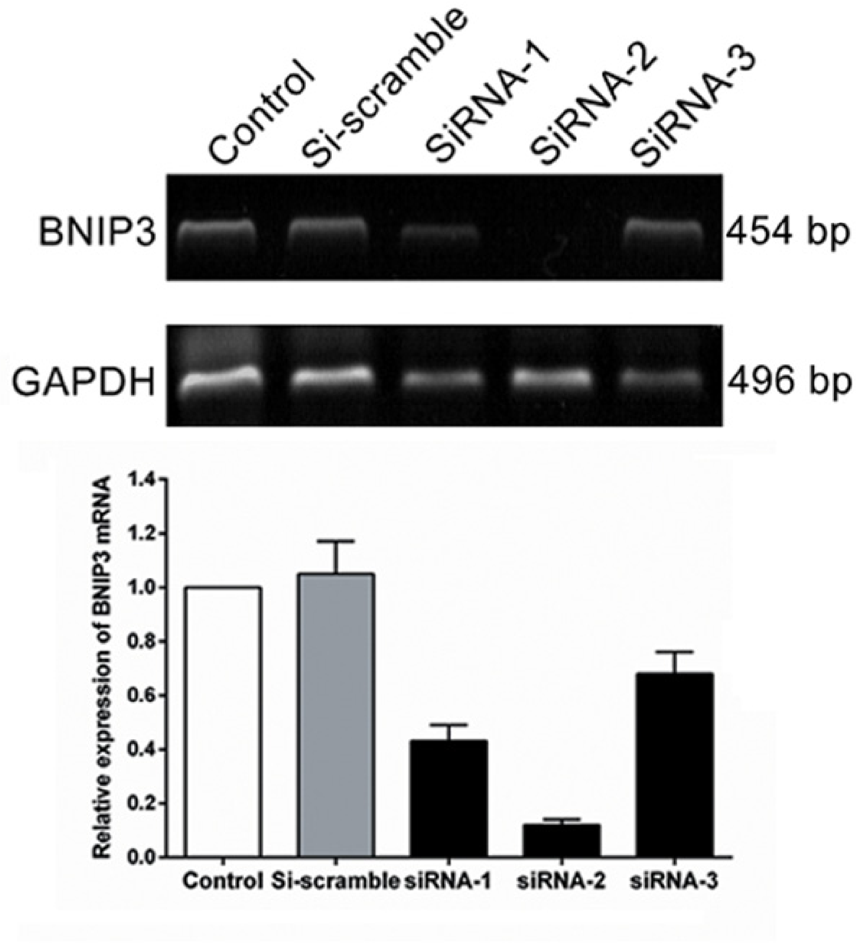

Supplementary Figure 1: Preliminary experiments demonstrated that the BNIP3-si1-homo-587 sequence exhibited the greatest efficiency with regard to the knockdown of BNIP3 expression.

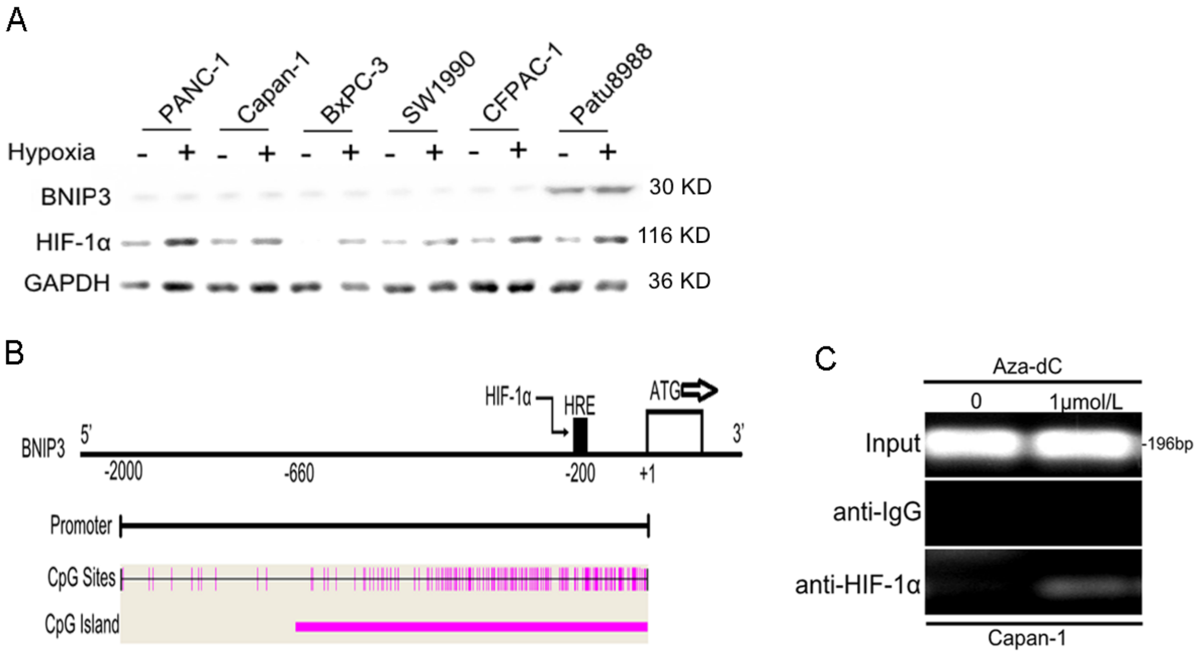

Supplementary Figure 2: Suppression of HIF-1α binding to the BNIP3 promoter by methylation. (A) The relative expression levels of HIF-1α and BNIP3 protein in pancreatic cancer cell lines (PANC-1, Capan-1, BXPC-3, SW1990, CFPAC-1, and Patu8988) under normoxic and hypoxic conditions were measured by Western blot assays. **(B)** Schematic showing the CpG island, CpG sites, and promoter region of the BNIP3 gene. The pink solid line represents a CpG island spanning from -660 bp to +1 bp of the transcription start site. The functional HRE was located within the CpG island. **(C)** ChIP assays were used to examined the binding of HIF-1α to the BNIP3 promoter in Aza-dC-treated Capan-1 cells. Specific anti-HIF-1α and/or control IgG were used in the immunoprecipitation experiments, whereas genomic DNA was used as input control. The images are representative of three independent experiments.

Supplementary Table 1: Relationship between BNIP3 expression and clinicopathological features in pancreatic cancer patients

Variables	Cases	BNIP3 expression		$\chi2$	P-value
		Negative	Positive		
Gender				0.259	0.611
Male	42	28	14		
Female	28	17	11		
Age (years)				3.434	0.064
≤65	41	26	15		
>65	29	19	10		
Tumor location[b]				0.804	0.370
Head	52	35	17		
Body and tail	18	10	8		
Differentiation				0.013	0.994
Well	8	5	3		
Moderate	17	11	6		
Poor	45	29	16		
Tstage				18.341	0.001[a]
T1	27	9	18		
T2	43	36	7		
N stage				5.240	0.022[a]
N0 (negative)	38	29	9		
N1 (positive)	32	16	16		
Clinical stage				9.990	0.002[a]
I	25	10	15		
II	45	35	10		

[a]$P<0.05$. [b]Head, body and tail refer to the location of the tumor in the pancreas.

Supplementary Table 2: Univariate and multivariate analysis of factors associated with overall survival in pancreatic cancer patients

Characteristics	Univariate analysis		Multivariate analysis	
	HR (95% CI)	*P*-value	HR (95% CI)	*P*-value
Gender	1.120 (0.675-1.859)	0.662		
Age(years)	1.115 (0.675-1.840)	0.672		
Tumor location	0.623 (0.351-1.105)	0.105		
Tumor size(cm)	1.046 (0.633-1.730)	0.860		
Differentiation	1.296 (0.899-1.867)	0.164		
Clinical stage	4.204 (2.395-7.378)	0.001	5.033 (2.506-10.110)	0.001
Lymph node metastasis	3.882 (2.284-6.599)	0.001	2.907 (1.467-5.762)	0.002
BNIP3 expression level	0.383 (0.224-0.655)	0.002	0.465 (0.240-0.904)	0.024
Caspase3 expression level	0.620 (0.365-1.055)	0.078		
Caspase9 expression level	0.648 (0.374-1.122)	0.121		
Bcl-2 expression level	2.091 (1.246-3.509)	0.005	2.055 (1.104-3.824)	0.023
Bax expression level	0.568 (0.344-0.938)	0.027	0.481 (0.275-0.843)	0.010

Supplementary Table 3: Correlation between the expression of BNIP3 and apoptosis associated proteins in pancreatic cancer tissues

Variables		BNIP3 expression		r	*P* value
		positive (n)	negative (n)		
Caspase 3	positive (n)	6	17	-0.141	0.246
	negative (n)	19	28		
Caspase 9	positive (n)	5	14	-0.120	0.324
	negative (n)	20	31		
Bcl-2	positive (n)	8	36	-0.476	0.001[a]
	negative (n)	17	9		
Bax	positive (n)	17	15	0.333	0.005 [a]
	negative (n)	8	30		

[a]$P<0.05$.
